# Lung Lesion Burden found on Chest CT as a Prognostic Marker in Hospitalized Patients with High Clinical Suspicion of COVID-19 Pneumonia: a Brazilian experience

**DOI:** 10.6061/clinics/2021/e3503

**Published:** 2021-11-23

**Authors:** Eduardo Kaiser Ururahy Nunes Fonseca, Antonildes Nascimento Assunção, Jose de Arimateia Batista Araujo-Filho, Lorena Carneiro Ferreira, Bruna Melo Coelho Loureiro, Daniel Giunchetti Strabelli, Lucas de Pádua Gomes de Farias, Rodrigo Caruso Chate, Giovanni Guido Cerri, Marcio Valente Yamada Sawamura, Cesar Higa Nomura

**Affiliations:** Hospital das Clinicas HCFMUSP, Faculdade de Medicina, Universidade de Sao Paulo, Sao Paulo, SP, BR.

**Keywords:** COVID-19, Pneumonia, Viral, Pandemics, Multidetector Computed Tomography, Diagnostic Imaging

## Abstract

**OBJECTIVE:**

To investigate the relationship between lung lesion burden (LLB) found on chest computed tomography (CT) and 30-day mortality in hospitalized patients with high clinical suspicion of coronavirus disease 2019 (COVID-19), accounting for tomographic dynamic changes.

**METHODS:**

Patients hospitalized with high clinical suspicion of severe acute respiratory syndrome coronavirus 2 (SARS-CoV-2) infection in a dedicated and reference hospital for COVID-19, having undergone at least one RT-PCR test, regardless of the result, and with one CT compatible with COVID-19, were retrospectively studied. Clinical and laboratory data upon admission were assessed, and LLB found on CT was semi-quantitatively evaluated through visual analysis. The primary outcome was 30-day mortality after admission. Secondary outcomes, including the intensive care unit (ICU) admission, mechanical ventilation used, and length of stay (LOS), were assessed.

**RESULTS:**

A total of 457 patients with a mean age of 57±15 years were included. Among these, 58% presented with positive RT-PCR result for COVID-19. The median time from symptom onset to RT-PCR was 8 days [interquartile range 6-11 days]. An initial LLB of ≥50% using CT was found in 201 patients (44%), which was associated with an increased crude at 30-day mortality (31% *vs.* 15% in patients with LLB of <50%, *p*<0.001). An LLB of ≥50% was also associated with an increase in the ICU admission, the need for mechanical ventilation, and a prolonged LOS after adjusting for baseline covariates and accounting for the CT findings as a time-varying covariate; hence, patients with an LLB of ≥50% remained at a higher risk at 30-day mortality (adjusted hazard ratio 2.17, 95% confidence interval 1.47-3.18, *p*<0.001).

**CONCLUSION:**

Even after accounting for dynamic CT changes in patients with both clinical and imaging findings consistent with COVID-19, an LLB of ≥50% might be associated with a higher risk of mortality.

## INTRODUCTION

The concern over multiple waves or spikes of the coronavirus disease 2019 (COVID-19) pandemic has created an unprecedented global challenge. Brazil was severely hit by COVID-19 following the first case on February 26, 2020, in São Paulo State (1). The country faces challenges, such as a shortage of testing capacity, significant diagnostic delays, and scarce health resources, such as hospital beds, personal protective equipment, and even physicians (2,3).

Reverse transcription-polymerase chain reaction (RT-PCR) assay is considered to be the gold standard for the diagnosis of COVID-19; however, a pooled analysis from seven studies revealed that false-negative rates are considerable and minimized when the testing occurs 1 week after the exposure (3 days after symptom onset) but remains high at 20% (4). More importantly, the studies showed that when the clinical pretest probability of infection is high, the post-test probability remains high even with a negative RT-PCR result, particularly 5 days after the onset of symptoms (4).

Chest computed tomography (CT) imaging has a high sensitivity for the early diagnosis of COVID-19 pneumonia using well-recognized classifications (5,6).

However, during the pandemic, chest CT went beyond the dichotomy of the diagnosis, whether or not it is suggestive of COVID-19. Most attending physicians want to know “and how bad is the disease extent.” Quantitative and semiquantitative chest CT analysis and relation to prognosis have been the subject of studies and discussion in radiologic literature, as it might lead to a more informative report (7). The estimation of the lung parenchyma involvement assessed in other coronaviruses was studied and described during the Middle East respiratory syndrome outbreaks using chest radiographs and chest CT, showing a correlation with poor prognosis (8,9). Of note, a CT lung score of 15/24, which roughly translates a lung lesion burden (LLB) of 60% (8), was associated with a higher mortality rate, a rationale for a similar approach in COVID-19. It is intuitive to imagine the reversed linear correlation between lung lesion burden and the prognosis of COVID-19, there are some data supporting this correlation (10-15); however, the available data regarding the dynamic changes of the CT during the disease’s natural course are still limited in the current study (16).

The primary outcome of this study was to investigate the prognostic value of LLB found on CT, accounting for its dynamic changes, at 30-day mortality in a cohort of hospitalized patients in Brazil with high clinical suspicion of COVID-19 pneumonia during the first wave of COVID-19. As secondary outcomes, we evaluated the impact of LLB on the length of stay (LOS), intensive care unit (ICU) admission, and the need for mechanical ventilation. We also assessed the association of RT-PCR results with both the primary and secondary outcomes.

## MATERIALS AND METHODS

This observational and retrospective study was performed in a single tertiary care medical center in Sao Paulo, Brazil, which was completely dedicated to the treatment of patients with high clinical suspicion of COVID-19 pneumonia and considered the largest center in the fight against COVID-19 in South America (2).

From March 16, 2020 to May 13, 2020, patients referred to the hospital with pneumonia and presenting a chest CT with a higher likelihood of COVID-19 according to the Radiological Society of North America (RSNA) statement on reporting chest CT findings related to COVID-19 (6) and at least one RT-PCR for COVID-19, regardless of the result, were included. Patients with atypical or negative CT findings (6) and those with negative RT-PCR results for COVID-19 but positive for other respiratory pathogens were excluded.

Our institutional review board approved this study, and the requirement for written informed consent was waived. The procedures were performed in accordance with the guidelines of the Declaration of Helsinki.

### Clinical and laboratory data

Clinical and laboratory data upon admission were collected from electronic health records.

The following clinical data were evaluated: age, sex, presence of comorbidities, including hypertension, diabetes, obesity, cancer, asthma, chronic obstructive pulmonary disease (COPD), cardiovascular disease (CVD), chronic kidney disease (CKD), and transplant recipient. The following laboratory data were collected: complete blood count, C-reactive protein, lactate dehydrogenase, creatinine, D-dimer, and bilirubin. The time between symptom onset, CT scanning, and relevant clinical events, such as hospital discharge or death, were also recorded. The primary outcome was the 30-day mortality, whereas the secondary outcomes were the LOS, ICU admission, and the need for mechanical ventilation.

RT-PCR assays were performed using the MagNA Pure 96 DNA and Viral NA Small Volume Kit (Roche Molecular Systems Inc.), whereas real-time PCR amplification was detected using the Charité protocol. Three samples were collected from each patient, one from each nostril, and one from the oropharynx. Patients with negative initial RT-PCR results and persistent clinical suspicion of COVID-19 were retested when clinically recommended (17).

### Chest CT protocol

CT examinations were performed using the multidetector CT scanners with 64-320 detector rows (Philips Brilliance 64 (multi-slice), Philips, USA; Canon Aquilion Prime and Aquilion One). All scans were obtained in a supine position during end-inspiration, with or without intravenous contrast material. The acquisition parameters for all CT scans were as follows: reconstructed slice thickness, 1 mm; voltage, 80-120 kVp; and automatic milliampere setting with a range of 10-440 mA. CT images were accessed through an integrated picture archiving and communication system.

### CT analysis

Two radiologists, EKUNF and DGS, both with 5 years of experience in interpreting chest imaging, blinded to clinical and laboratory data, reviewed all chest CT images independently in a clinical Picture Archiving and Diagnostic System workstation. They evaluated image quality and classified the CT findings according to the specific RSNA Statement (6) and visually quantified the global LLB in a dichotomized fashion as <50% or ≥50% of the lung parenchyma involvement as the current most used cut-off for severe COVID-19 extent on CT (18). Any divergence between the radiologists was resolved by consensus and verified by a more experienced radiologist, MVYS with 10 years of experience.

### Statistical analyses

Data are expressed as means±standard deviations or medians and interquartile ranges (IQR) for continuous variables. Normality assumption was assessed graphically, such as the QQ plot, and confirmed using the Shapiro-Wilk test. Differences in baseline characteristics and outcomes between groups according to LLB were compared using the χ2 or Fisher exact test for categorical variables and Student’s t-test or Mann-Whitney U test for continuous variables, when appropriate.

Survival analysis with the dependent variable as the time to mortality was performed to investigate the prognostic impact of LLB. Hospital admission was set as time zero, and patients were censored on the day of hospital discharge, at 30-day mortality, or data freeze (May 13, 2020), whichever came first. We then created a Cox proportional hazards model adjusting for baseline risk factors, including age >60 years, diabetes, obesity, smoking, COPD, CVD, CKD, and cancer (19,20), and accounting for LLB as a time-varying covariate. We further added RT-PCR status to the final model. All statistical analyses were performed using the R version 4.0.5 (R Foundation for Statistical Computing, Vienna, Austria) and a *p*-value <0.05 was considered statistically significant.

## RESULTS

### Baseline patient characteristics

**Figure 1:**
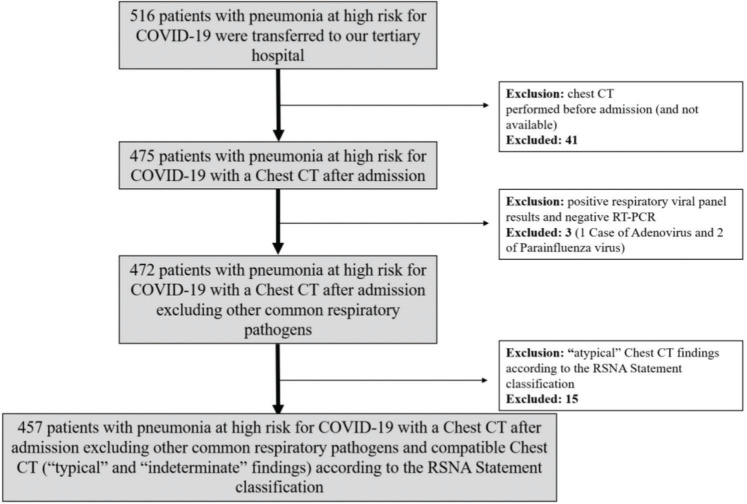
Flow diagram of patients’ enrollment.

During the study period, 516 patients with high clinical suspicion of COVID-19 pneumonia were transferred to our tertiary hospital. Of these, 51 patients (10%) were excluded: 41 with CT performed before admission and not available; 15 with an atypical CT pattern; and 3 with positive respiratory viral panel and negative RT-PCR results (1 and 2 cases of adenovirus and parainfluenza virus infections, respectively) (Figure 1). The remaining cohort included 457 patients presenting pneumonia with typical (77%) or indeterminate (23%) imaging features according to the RSNA statement classification (6). The baseline characteristics of these patients are shown in Tables 1, 2, and 3. The mean age was 57±15 years, 48% were male, and 76% had at least one baseline comorbidity. RT-PCR was positive in 58% of the patients, with a median time from symptoms to RT-PCR of 8 days (IQR 6-11 days). When the patients’ characteristics were stratified according to the RT-PCR results, cancer and CVD were more common in patients with positive RT-PCR results, while the remaining characteristics did not differ (Table 2).

### CT findings

Most patients underwent CT within 24h upon admission, with a median time from symptom onset to CT of 8 days (IQR 6-12 days) (Table 1). LLB found on CT was <50% in 256 patients (56%), whereas it was ≥50% in 201 patients (44%). Typical findings were more prevalent in patients with LLB <50% than in those with LLB ≥50% (88% *vs.* 64%, *p*<0.001) and positive RT-PCR results (64% *vs.* 50%, *p*=0.003). Except for CKD, the prevalence of comorbidities was similar in patients with LLB found on CT of < 50% and ≥50%.

### Primary outcome

**Figure 2:**
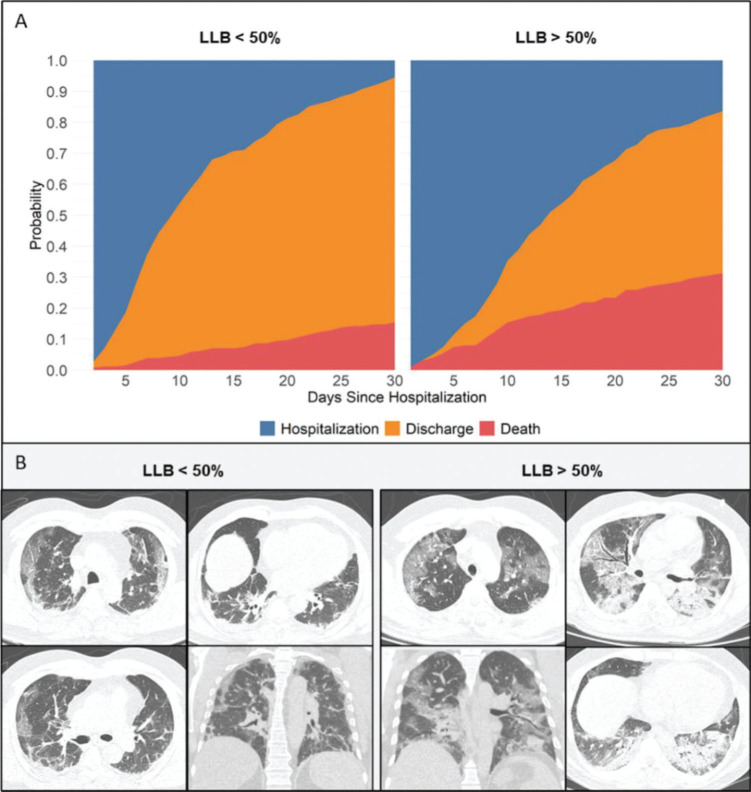
A) Cumulative incidence plots for the three possible outcomes (mortality, discharged, and continued hospitalization) according to the LLB. B) Chest CT illustrating an LLB of <50% (left images) and LLB of >50% (right images).

**Figure 3:**
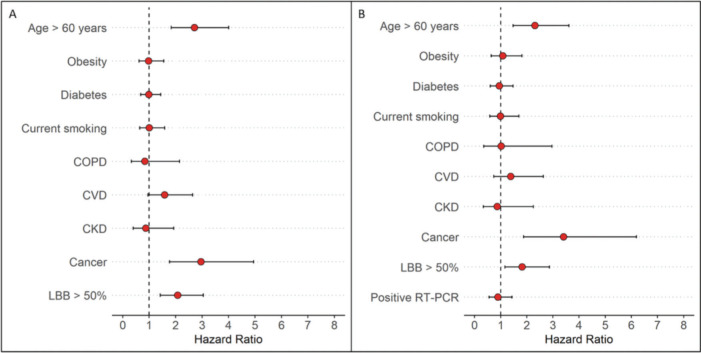
Forest plot of Cox proportional hazard multivariable modeling on survival for hospitalized patients with COVID-19 after adjusting for baseline covariates and accounting for the CT findings as a time-varying covariate. A) Not including RT-PCR in the model. B) After adding RT-PCR in the model.

At the time of data freezing, 99 patients (22%) died, 302 were discharged (66%), and 56 remained hospitalized (12%). The median time to event was 13 days (IQR, 8-27 days). Figure 2 displays the cumulative incidence plots for the three possible outcomes (mortality, discharge, and continued hospitalization) according to the LLB. An LLB of ≥50% was associated with less frequent discharge and an increased crude at 30-day mortality (31% *vs*. 15% in patients with LLB <50%, *p*<0.001). Of note, among the 52 patients with negative RT-PCR and indeterminate CT findings, there were 13 deaths within 30 days of hospitalization. All patients presented with an LLB of ≥50% found on CT. In a Cox proportional hazards regression model after adjusting for baseline covariates and accounting for the CT findings as a time-varying covariate, patients with an LLB of ≥50% presented increased 30-day mortality (adjusted hazard ratio [HR] 2.17, 95% confidence interval [CI] 1.47-3.18, *p*<0.001). After adding RT-PCR in the model, an LLB of ≥50% remained associated with an increased risk of death (adjusted HR 1.82, 95% CI 1.16-2.86, *p*=0.008), whereas the RT-PCR result was not associated with this primary outcome (Figure 3).

### Secondary outcomes

When comparing patients with an LLB of ≥50%, the first subgroup had a longer LOS (mean LOS of 15 days×9 days), more often were admitted to the ICU (63%×49%), and more usually needed mechanical ventilation (33%×27%) than those with an LLB of <50% (Table 3).

Considering the RT-PCR results, when arranging the patients according to the RT-PCR results, patients with positive RT-PCR results had an increased crude 30-day mortality (70/264) when compared with those with negative RT-PCR results (29/193). Furthermore, patients with positive RT-PCR results had a longer LOS and were often admitted to the ICU than those with negative RT-PCR results. The need for mechanical ventilation did not differ between the subgroups. These results are presented in Table 2.

## DISCUSSION

In this cohort of 457 patients with high clinical suspicion of COVID-19 pneumonia and referred to a COVID-19 dedicated tertiary hospital, we found that a greater extent of pulmonary involvement (LLB of ≥50%) was associated with a two-fold increase in 30-day mortality when accounting for baseline differences and CT as a time-varying covariate. In addition to an LLB of ≥50%, the presence of cancer and age >60 years were also associated with greater mortality at 30 days in the multivariate analysis, accounting for baseline differences and CT as a time-varying covariate; however, this was not only subject to change after adding the RT-PCR results to the model.

Our data also showed that patients with an LLB of ≥50% had a prolonged LOS, more often were admitted to the ICU, and usually needed mechanical ventilation.

During the study period (first wave of pandemic), delay for testing occurred among most patients (25^th^ percentile time from symptoms to RT-PCR of 6 days), with more than 40% of patients presenting negative results, despite the suggestive clinical and imaging findings. Of note, the effect of LLB on 30-day mortality remained comparable when adjusted for RT-PCR results.

During the first COVID-19 wave in Brazil, there was a limited testing capacity (3), which might have led to delays and false-negative results in the study (21-23). This is supported by the high pretest probability and typical CT findings (nearly three-quarters) of our cohort. In a similar context, a Dutch experience in the emergency department during the first COVID-19 wave of six centers has been recently reported (24). Of the 1070 patients, with a median time from symptoms to testing of 7 days, only 50% had a positive RT-PCR result. Using a clinical standard reference, the study showed that typical CT findings (COVID-19 Reporting and Data System 4 and 5) were comparable with RT-PCR results to reliably diagnose COVID-19 (area under the curve for both 0.87) (24).

Many investigators have shown the potential association between initial CT findings, primarily the extent of pulmonary involvement, and adverse outcomes in patients with COVID-19 pneumonia (10,11,13,15,25-29). Clinical endpoints and magnitude of the association with CT findings varied greatly between these studies, as did the extent of involvement used as the cut-off point. In addition, different statistical methodologies were applied, such as logistic regression, which excludes patients who were still hospitalized, including longer-staying patients who were generally older and with more comorbidities, and the standard Cox regression models do not account for CT findings as time-dependent confounding, both impacting risk estimates and leading to possible biases (30). Our study first confirmed the association of the initial CT on mortality using time-to-event models and accounting for the observed time dynamics of CT findings over time. We also observed that an initial CT with an LLB of ≥50% was associated with a prolonged LOS, more often ICU admission, and usually needed mechanical ventilation.

Of note, 50% of patients underwent initial CT between 6 and 12 days after symptom onset. This time window is similar to that reported as the peak of the extent of CT abnormalities in a longitudinal study (16). Thus, this interval might be considered in clinical practice for requesting a chest CT for the prognostication of COVID-19.

It is important to acknowledge that, although all patients presented pneumonia with high clinical pre-test probability for COVID-19, 52 patients presented indeterminate CT findings and negative RT-PCR results, which can be argued as a “less possible” COVID-19 diagnosis. However, all deaths in this subgroup occurred in patients with LLB of ≥50% found on CT, in which typical findings of rounded or sparse peripheral opacities are usually replaced by more diffuse opacities (indeterminate appearance) as the disease progresses (16).

A subgroup analysis showed poorer outcomes in the patients with positive RT-PCR, and we found a higher crude 30 day-mortality in this subset of patients, in which they were more often admitted to the ICU and had a prolonged LOS compared with those with negative RT-PCR results. As discussed above, those with negative RT-PCR results, at least in part, should show false-negative laboratory results, as most of our patients were tested after 6 days of symptoms; hence, RT-PCR is prone to lose its sensitivity (4). There is no single answer as to why patients with positive RT-PCR results show worse outcomes, while some insights from recent literature (31) demonstrate that, in patients with critical and severe COVID-19, the viral load was higher, which translated more often into persistent positive RT-PCR results. As the viral load was not quantified in our study, this might be a possible indirect inference of a higher viral load. The absence of a correlation between the RT-PCR results and prognostication after multivariate analysis for the CT findings as a time-varying covariate point that the dichotomous RT-PCR result *per se* is not a factor directly associated with prognosis, as the viral load might be. This question remains open, and further research focused on the correlation between chest CT, viral load, and prognosis of COVID-19 are warranted to address this issue.

Another limitation of this study is that the results represent the first COVID-19 wave in Brazil with a high incidence, high clinical pretest likelihood, and severely ill individuals referred to a tertiary hospital, which precludes generalization to other clinical settings.

## CONCLUSION

In a similar clinical scenario of patients with flu-like syndrome and with a chest CT with findings consistent with COVID-19, our data suggest that even after accounting for CT dynamic changes, an LLB of ≥50% might be associated with a higher risk of mortality regardless of the RT-PCR result.

## AUTHOR CONTRIBUTIONS

All the authors conceived and designed the study. Fonseca EKUN, Strabelli DG, Farias LPG, Ferreira LC and Sawamura MVY collected the data. Fonseca EKUN, Loureiro BMC, Assunção Junior AN, Araujo-Filho JAB, Chate RC, Cerri GG, Sawamura MVY and Nomura CH analyzed the data. All authors helped in writing the manuscript. All authors contributed to the manuscript revisions, approved the final version of the manuscript, and agreed to hold accountable for the content therein.

## Figures and Tables

**Table 1:** Baseline characteristics and outcomes of admitted patients.

	All patients* (n=457)
Clinical variables	
Age, years	57±15
Male sex, n (%)	220 (48)
Smoking, n (%)	71 (16)
Obesity, n (%)	95 (21)
Hypertension, n (%)	218 (48)
Diabetes, n (%)	150 (33)
Cardiovascular disease, n (%)	45 (10)
Asthma, n (%)	15 (3)
COPD, n (%)	14 (3)
Chronic kidney disease, n (%)	27 (6)
Cancer, n (%)	29 (6)
Transplant recipient, n (%)	14 (3)
Any comorbidity, n (%)	349 (76)
Oxygen saturation, %	92 [88-94]
Respiratory rate	25 [22-30]
Fever, n (%)	284 (62)
Symptoms to RT-PCR, days	8 [6-11]
Admission to RT-PCR, days	0 [0-1]
Positive RT-PCR	264 (58)
Laboratory values	
Hemoglobin, mg/L	13±2
Hematocrit, %	39±6
Platelet, 10^9^/L	222 [171-288]
NL ratio	6.2 [3.6-10.9]
LDH, UI/L	402 [303-522]
CRP, mg/L	137 [73-218]
D-dimer, ng/mL	1371 [710-3307]
Bilirubin (total), mg/dL	0.42 [0.26-0.61]
Creatinine, mg/dL	0.9 [0.71-1.42]
CT findings	
Symptoms to CT, days	8 [6-12]
Admission to CT, days	0 [0-1]
Typical CT findings, n (%)	353 (77)
Outcomes	
30-day mortality, n (%)	99 (22)
ICU admission, n (%)	260 (57)
Mechanical ventilation, n (%)	140 (31)
LOS, days	12 [7-20]

COPD, chronic pulmonary obstructive disease; NL ratio, neutrophil-to-lymphocyte ratio; LDH, lactate dehydrogenase; CRP, C-reactive protein; ICU, intensive care unit; LOS, length of stay.

**Table 2:** Baseline characteristics and outcomes of admitted patients, stratified by RT-PCR result.

	Negative RT-PCR* (n=193)	Positive RT-PCR* (n=264)	*p*-value
Age, years	56±16	57±15	0.181
Male sex, n (%)	98 (51)	122 (46)	0.384
Obesity, n (%)	39 (20)	56 (21)	0.885
Hypertension, n (%)	84 (44)	134 (51)	0.151
Diabetes, n (%)	59 (31)	91 (35)	0.438
Cardiovascular disease, n (%)	12 (6)	33 (13)	**0.039**
Asthma, n (%)	5 (3)	10 (4)	0.657
COPD, n (%)	8 (4)	6 (2)	0.383
Chronic kidney disease, n (%)	10 (5)	17 (6)	0.717
Cancer, n (%)	5 (3)	24 (9)	**0.009**
Transplant recipient, n (%)	3 (2)	7 (3)	0.640
Smoking, n (%)	33 (17.1)	38 (14.4)	0.511
Symptoms to RT-PCR, days	8 [6-12]	8 [5-11]	0.186
Admission to RT-PCR, days	0 [0-1]	0 [0-1]	0.283
Symptoms to CT, days	8 [7-13]	7 [5-11]	0.180
Typical CT findings, n (%)	141 (73)	212 (80)	0.087
Outcomes			
30-day mortality, n (%)	29 (15)	70 (27)	**0.004**
ICU admission, n (%)	95 (49)	165 (63)	**0.006**
Mechanical ventilation, n (%)	52 (27)	88 (33)	0.174
LOS, days	9 [6-14]	15 [8-24]	**<0.001**

COPD, chronic pulmonary obstructive disease; NL ratio, neutrophil-to-lymphocyte ratio; LDH, lactate dehydrogenase; CRP, C-reactive protein; ICU, intensive care unit; LOS, length of stay.

**Table 3:** Baseline and outcomes characteristics of admitted patients, stratified by LLB result.

	LLB <50%* (n=256)	LLB ≥50%* (n=201)	*p*-value
Age, years	56±16	57±15	0.782
Male sex, n (%)	135 (53)	85 (42)	**0.034**
Smoking, n (%)	39 (15)	32 (16)	0.943
Obesity, n (%)	52 (20)	43 (21)	0.868
Hypertension, n (%)	128 (50)	90 (45)	0.310
Diabetes, n (%)	92 (36)	58 (29)	0.134
Cardiovascular disease, n (%)	30 (12)	15 (8)	0.175
Asthma, n (%)	12 (5)	3 (2)	0.101
COPD, n (%)	8 (3)	6 (3)	0.999
Chronic kidney disease, n (%)	21 (8)	6 (3)	**0.032**
Cancer, n (%)	20 (8)	9 (5)	0.208
Transplant recipient, n (%)	10 (4)	4 (2)	0.365
Any comorbidity, n (%)	198 (77)	151 (75)	0.657
Symptoms to RT-PCR, days	8 [5-11]	8 [6-11]	0.102
Admission to RT-PCR, days	0 [0-1]	1 [0-1]	**0.049**
Positive RT-PCR	164 (64)	100 (50)	**0.003**
Symptoms to CT, days	7 [5-11]	8 [6-12]	0.070
Admission to CT, days	0 [0-1]	0 [0-1]	0.454
Typical CT findings, n (%)	225 (88)	128 (64)	**<0.001**
Outcomes			
30-day mortality, n (%)	37 (15)	62 (31)	**<0.001**
ICU admission, n (%)	104 (41)	156 (78)	**<0.001**
Mechanical ventilation, n (%)	38 (15)	102 (51)	**<0.001**
LOS, days	10 [6-18]	14 [9-23]	**<0.001**

COPD, chronic pulmonary obstructive disease; NL ratio, neutrophil-to-lymphocyte ratio; LDH, lactate dehydrogenase; CRP, C-reactive protein; ICU, intensive care unit; LOS, length of stay.

## References

[1] Chate RC, Fonseca EKUN, Passos RBD, Teles GBDS, Shoji H, Szarf G (2020). Presentation of pulmonary infection on CT in COVID-19: initial experience in Brazil. J Bras Pneumol.

[2] Tan BS, Dunnick NR, Gangi A, Goergen S, Jin ZY, Neri E (2021). RSNA International Trends: A Global Perspective on the COVID-19 Pandemic and Radiology in Late 2020. Radiology.

[3] Hasell J, Mathieu E, Beltekian D, Macdonald B, Giattino C, Ortiz-Ospina E (2020). A cross-country database of COVID-19 testing. Sci Data.

[4] Kucirka LM, Lauer SA, Laeyendecker O, Boon D, Lessler J (2020). Variation in False-Negative Rate of Reverse Transcriptase Polymerase Chain Reaction-Based SARS-CoV-2 Tests by Time Since Exposure. Ann Intern Med.

[5] Fang Y, Zhang H, Xie J, Lin M, Ying L, Pang P (2020). Sensitivity of Chest CT for COVID-19: Comparison to RT-PCR. Radiology.

[6] Simpson S, Kay FU, Abbara S, Bhalla S, Chung JH, Chung M (2020). Radiological Society of North America Expert Consensus Document on Reporting Chest CT Findings Related to COVID-19: Endorsed by the Society of Thoracic Radiology, the American College of Radiology, and RSNA. Radiol Cardiothorac Imaging.

[7] Chen A, Karwoski RA, Gierada DS, Bartholmai BJ, Koo CW (2020). Quantitative CT Analysis of Diffuse Lung Disease. Radiographics.

[8] Das KM, Lee EY, Al Jawder SE, Enani MA, Singh R, Skakni L (2015). Acute Middle East Respiratory Syndrome Coronavirus: Temporal Lung Changes Observed on the Chest Radiographs of 55 Patients. AJR Am J Roentgenol.

[9] Ajlan AM, Ahyad RA, Jamjoom LG, Alharthy A, Madani TA (2014). Middle East respiratory syndrome coronavirus (MERS-CoV) infection: chest CT findings. AJR Am J Roentgenol.

[10] Charpentier E, Soulat G, Fayol A, Hernigou A, Livrozet M, Grand T (2021). Visual lung damage CT score at hospital admission of COVID-19 patients and 30-day mortality. Eur Radiol.

[11] Zhou S, Chen C, Hu Y, Lv W, Ai T, Xia L (2020). Chest CT imaging features and severity scores as biomarkers for prognostic prediction in patients with COVID-19. Ann Transl Med.

[12] Colombi D, Villani GD, Maffi G, Risoli C, Bodini FC, Petrini M (2020). Qualitative and quantitative chest CT parameters as predictors of specific mortality in COVID-19 patients. Emerg Radiol.

[13] Raoufi M, Safavi Naini SAA, Azizan Z, Jafar Zade F, Shojaeian F, Ghanbari Boroujeni M (2020). Correlation between Chest Computed Tomography Scan Findings and Mortality of COVID-19 Cases; a Cross sectional Study. Arch Acad Emerg Med.

[14] Francone M, Iafrate F, Masci GM, Coco S, Cilia F, Manganaro L (2020). Chest CT score in COVID-19 patients: correlation with disease severity and short-term prognosis. Eur Radiol.

[15] Besutti G, Ottone M, Fasano T, Pattacini P, Iotti V, Spaggiari L (2021). The value of computed tomography in assessing the risk of death in COVID-19 patients presenting to the emergency room. Eur Radiol.

[16] Wang Y, Dong C, Hu Y, Li C, Ren Q, Zhang X (2020). Temporal Changes of CT Findings in 90 Patients with COVID-19 Pneumonia: A Longitudinal Study. Radiology.

[17] World Health Organization (WHO) (2020). Laboratory testing for coronavirus disease 2019 (COVID-19) in suspected human cases. World Health Organization.

[18] Wu Z, McGoogan JM (2020). Characteristics of and Important Lessons from the Coronavirus Disease 2019 (COVID-19) Outbreak in China: Summary of a Report of 72314 Cases from the Chinese Center for Disease Control and Prevention. JAMA.

[19] Grasselli G, Greco M, Zanella A, Albano G, Antonelli M, Bellani G (2020). Risk Factors Associated With Mortality Among Patients With COVID-19 in Intensive Care Units in Lombardy, Italy. JAMA Intern Med.

[20] Richardson S, Hirsch JS, Narasimhan M, Crawford JM, McGinn T, Davidson KW (2020). Presenting Characteristics, Comorbidities, and Outcomes Among 5700 Patients Hospitalized With COVID-19 in the New York City Area. JAMA.

[21] Lv DF, Ying QM, Weng YS, Shen CB, Chu JG, Kong JP (2020). Dynamic change process of target genes by RT-PCR testing of SARS-Cov-2 during the course of a Coronavirus Disease 2019 patient. Clin Chim Acta.

[22] Li Y, Yao L, Li J, Chen L, Song Y, Cai Z (2020). Stability issues of RT-PCR testing of SARS-CoV-2 for hospitalized patients clinically diagnosed with COVID-19. J Med Virol.

[23] Long C, Xu H, Shen Q, Zhang X, Fan B, Wang C (2020). Diagnosis of the Coronavirus disease (COVID-19): rRT-PCR or CT?. Eur J Radiol.

[24] Schalekamp S, Bleeker-Rovers CP, Beenen LFM, Quarles van Ufford HME, Gietema HA, Stöger JL (2021). Chest CT in the Emergency Department for Diagnosis of COVID-19 Pneumonia: Dutch Experience. Radiology.

[25] Timaran-Montenegro DE, Torres-Ramírez CA, Morales-Jaramillo LM, Mateo-Camacho YS, Tapia-Rangel EA, Fuentes-Badillo KD (2021). Computed Tomography-based Lung Residual Volume and Mortality of Patients With Coronavirus Disease-19 (COVID-19). J Thorac Imaging.

[26] Colombi D, Bodini FC, Petrini M, Maffi G, Morelli N, Milanese G (2020). Well-aerated Lung on Admitting Chest CT to Predict Adverse Outcome in COVID-19 Pneumonia. Radiology.

[27] Ruch Y, Kaeuffer C, Ohana M, Labani A, Fabacher T, Bilbault P (2020). CT lung lesions as predictors of early death or ICU admission in COVID-19 patients. Clin Microbiol Infect.

[28] Khosravi B, Aghaghazvini L, Sorouri M, Naybandi Atashi S, Abdollahi M, Mojtabavi H (2021). Predictive value of initial CT scan for various adverse outcomes in patients with COVID-19 pneumonia. Heart Lung.

[29] Abbasi B, Akhavan R, Ghamari Khameneh A, Zandi B, Farrokh D, Pezeshki Rad M (2021). Evaluation of the relationship between inpatient COVID-19 mortality and chest CT severity score. Am J Emerg Med.

[30] Wolkewitz M, Lambert J, von Cube M, Bugiera L, Grodd M, Hazard D (2020). Statistical Analysis of Clinical COVID-19 Data: A Concise Overview of Lessons Learned, Common Errors and How to Avoid Them. Clin Epidemiol.

[31] Rabaan AA, Tirupathi R, Sule AA, Aldali J, Mutair AA, Alhumaid S (2021). Viral Dynamics and Real-Time RT-PCR Ct Values Correlation with Disease Severity in COVID-19. Diagnostics (Basel).

